# Iatrogenic fornix rupture caused during retrograde manipulation of the ureter: a case report

**DOI:** 10.1186/1757-1626-1-320

**Published:** 2008-11-17

**Authors:** Andreas Bannowsky

**Affiliations:** 1 Dept. of Urology, Ev.-Luth. Diakonissen Hospital Flensburg, Germany

## Abstract

Iatrogenic fornix rupture caused during retrograde manipulation of the ureter is a rather rare or rarely diagnosed phenomenon. A 22 year-old female patient presented with a fornix rupture following endoscopic ureteral stone extraction under uretero-renoscopy, the rupture having become symptomatic two days later.

## Background

The fornix rupture, a special form of the obstructive nephropathy, presents a rather rare and/or rarely diagnosed phenomenon. We report of a 22 year old female patient who was transfered to us after suspicion of a fornix rupture two days after receiving ureterorenoscopic extraction of an ureteral stone.

## Case presentation

An ureterorenoscopy with extraction of an ureteral stone was performed on a 22 year old female patient after two unsuccessful attempts of extracorporeal shock wave lithotripsy. Dilatation of the upper urinary tract as well as peri- or intrarenal urinary extravasation did not exist before endoscopic stone removal.

**Figure 1:**
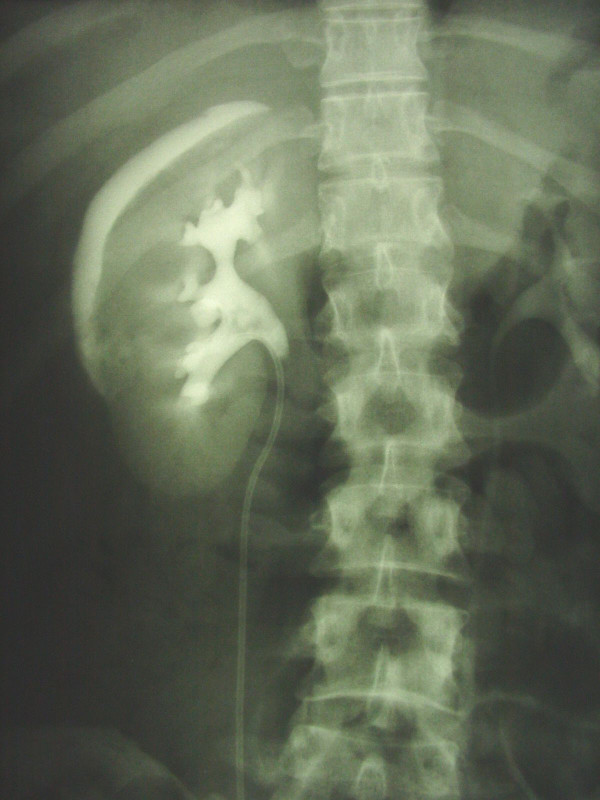
Abdominal plain radiograph with sickle-shaped perirenal contrast medium extravasation.

**Figure 2:**
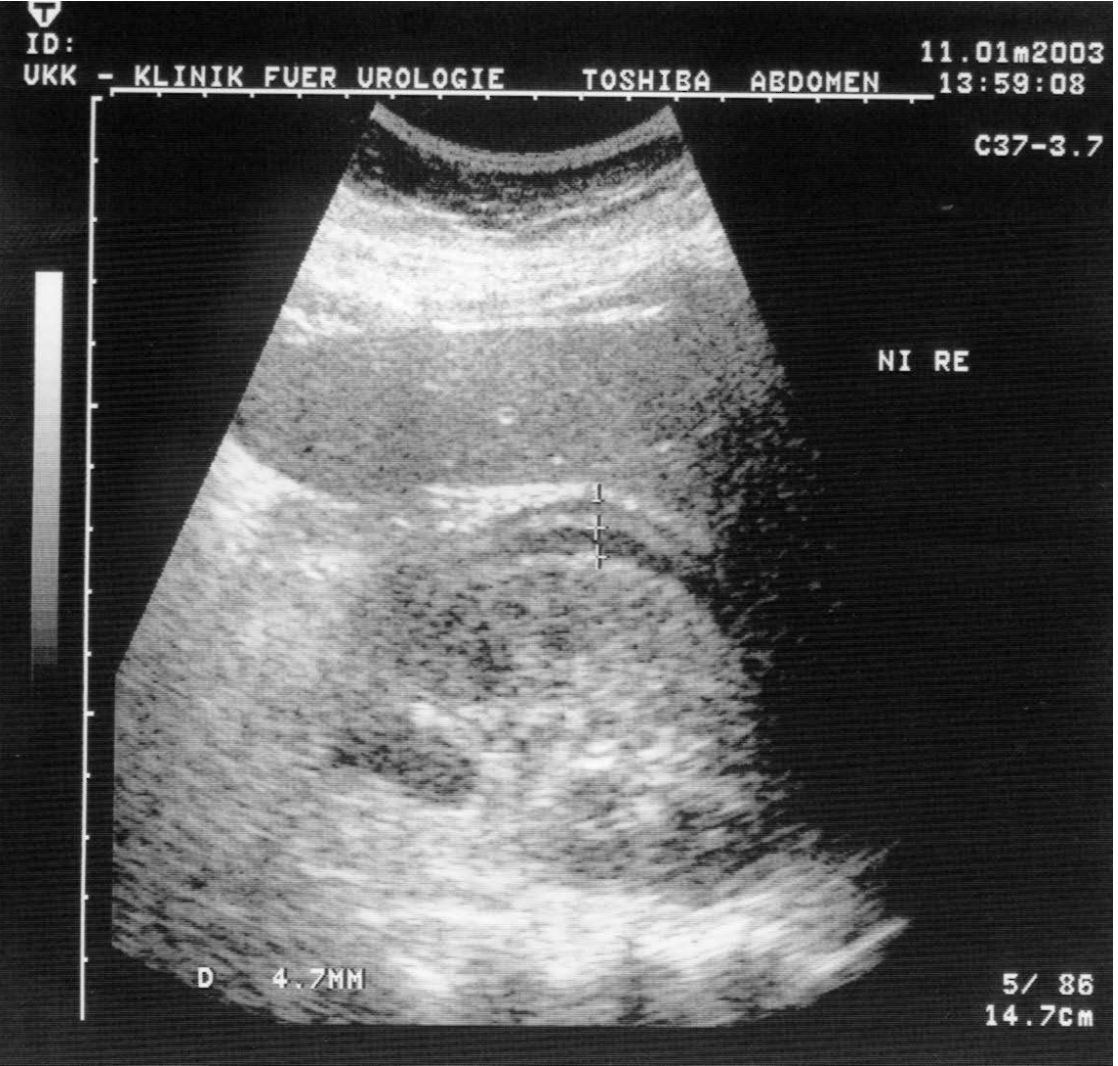
Ultrasound image of the right kidney with perirenal fluid extravasation within the Gerota's fascia.

On the postoperative abdominal plain radiograph a perirenal sickle-shaped contrast medium paravasation was shown (Fig. 1). During sonographic controls an extravasation existed within the Gerota's fascia (Fig. 2). The upper urinary tract and renal pelvis weren't dilated with the double-pigtail-ureterstent in correct position.

On the second postoperative day the patient developed progredient symptoms with flankpain spraying into the right upper and lower abdomen. Therefore she was transfered to our clinic for further diagnostics and therapy.

**Figure 3:**
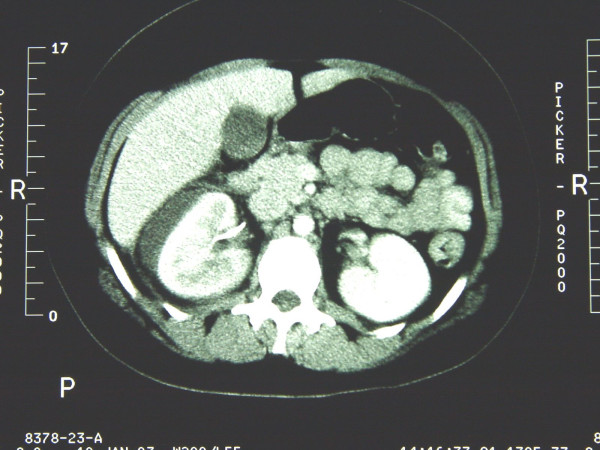
Abdominal CT scan with perirenal extravasation without evidence of abscess formation.

An urinary tract infection with *Escherichia coli *was detected in the urine culture. In the abdominal CT scan the previously sonographically diagnosed paravasation was found without signs of abscess formation (Fig. 3). A conservative analgetic and antibiotic therapy was initiated. The symptoms improved rapidly within the next 3 days with significant reduction in size of the extravasation during the regularly performed sonographic controls. Due to these results an operative intervention wasn't necessary. The patient was discharged asymptomatically from the hospital after 5 days.

## Discussion

Every supravesical obstruction might be a possible reason for developing a fornix rupture. The most common causes are obstruent ureter stones. 2–3% of all colics caused by stones are supposed to be followed by a fornix rupture [1]. The second most common cause is the iatrogenic restriction of the ureter, i.e. due to gynecological surgeries. Further possible causes are restrictions through obstruent tumors or a traumatic event with chronic obstruction [2].

The iatrogenic caused fornix rupture through retrograde ureter manipulation is a rather rare or rarely diagnosed phenomenon, because after disobstruction of the upper urinary tract the rupture remains mostly asymptomatic. The urinary extravasation will be reabsorbed through the lymphatic system in the perirenal fat [3].

Requirement for a mild process, which justifies a conservative treatment, is the sterility of the urine. On the other hand, if the urine is infected, the risk of febrile to septic pyelonephritis with perirenal formation of an abscess increases dramatically. Approximately 10% of all perirenal abscesses are caused by urinay extravasation due to fornix ruptures [4]. In these cases an operative treatment should be performed immediately.

Regular postoperative sonographic controls are absolutely necessary to diagnose an urinary extravasation in time to induce an appropriate therapy, especially if additive febrile symptomatic has become evident. Even in the case of a non-dilated upper urinary tract with a correct placement of the ureterstent after endoscopic uretermanipulation, this approach is recommended as well.

## Consent

Written informed consent was obtained from the patient for publication of this case report and accompanying images. A copy of the written consent is available for review by the Editor-in-Chief of this journal.

## Competing interests

The author declares that they have no competing interests.

## Authors' contributions

AB analyzed and interpreted the patient data, and was the major contributor in writing the manuscript. He approved the final manuscript.
